# High levels of pericoronary adipose tissue inflammation are associated with coronary atherosclerosis independent of epicardial adipose tissue volume in patients with chronic coronary syndrome

**DOI:** 10.1093/ehjimp/qyaf079

**Published:** 2025-06-10

**Authors:** Hiroki Yamaura, Kenichiro Otsuka, Hirotoshi Ishikawa, Kana Hojo, Kotaro Matsumoto, Naoki Fujisawa, Akihiro Okamoto, Tomohiro Yamaguchi, Shunsuke Kagawa, Takenobu Shimada, Atsushi Shibata, Asahiro Ito, Takanori Yamazaki, Kenei Shimada, Noriaki Kasayuki, Daiju Fukuda

**Affiliations:** Department of Cardiovascular Medicine, Osaka Metropolitan University Graduate School of Medicine, Osaka 545-8585, Japan; Department of Cardiovascular Medicine, Osaka Metropolitan University Graduate School of Medicine, Osaka 545-8585, Japan; Department of Cardiovascular Medicine, Kashibaseiki Hospital, Kashiba, Nara 639-0252, Japan; Department of Cardiovascular Medicine, Osaka Metropolitan University Graduate School of Medicine, Osaka 545-8585, Japan; Department of Cardiovascular Medicine, Osaka Metropolitan University Graduate School of Medicine, Osaka 545-8585, Japan; Department of Cardiovascular Medicine, Osaka Metropolitan University Graduate School of Medicine, Osaka 545-8585, Japan; Department of Cardiovascular Medicine, Osaka Metropolitan University Graduate School of Medicine, Osaka 545-8585, Japan; Department of Cardiovascular Medicine, Osaka Metropolitan University Graduate School of Medicine, Osaka 545-8585, Japan; Department of Cardiovascular Medicine, Osaka Metropolitan University Graduate School of Medicine, Osaka 545-8585, Japan; Department of Cardiovascular Medicine, Osaka Metropolitan University Graduate School of Medicine, Osaka 545-8585, Japan; Department of Cardiovascular Medicine, Osaka Metropolitan University Graduate School of Medicine, Osaka 545-8585, Japan; Department of Cardiovascular Medicine, Osaka Metropolitan University Graduate School of Medicine, Osaka 545-8585, Japan; Department of Cardiovascular Medicine, Osaka Metropolitan University Graduate School of Medicine, Osaka 545-8585, Japan; Department of Cardiovascular Medicine, Kashibaseiki Hospital, Kashiba, Nara 639-0252, Japan; Department of Cardiovascular Medicine, Kashibaseiki Hospital, Kashiba, Nara 639-0252, Japan; Department of Cardiovascular Medicine, Osaka Metropolitan University Graduate School of Medicine, Osaka 545-8585, Japan

**Keywords:** atherosclerosis, inflammation, adipose tissue, computed tomography, calcification, pericoronary artery adipose tissue

## Abstract

**Aims:**

This study aimed to assess clinical risks and coronary atherosclerotic burden in patients with chronic coronary syndrome (CCS) stratified by pericoronary artery adipose tissue (PCAT) composition and epicardial adipose tissue volume (EAV).

**Methods and results:**

We retrospectively included 410 CCS patients who underwent coronary computed tomography angiography. Patients were divided into four groups based on an EAV index ≥ 73.5 mL/mm^2^ and PCAT attenuation (PCATA) in the right coronary artery (PCATA_RCA_) ≥ −76.6 HU (above median); Groups A (low EAV index and low PCATA_RCA_), B (low EAV index and high PCATA_RCA_), C (high EAV index and low PCATA_RCA_), and D (high EAV index and high PCATA_RCA_). Multivariable models assessed the relative risk of coronary artery calcium score (CACS) > 400 and coronary artery disease (CAD), and predictors of coronary plaque volume. The log-transformed CACS increased progressively, with Group D showing the highest values. Group D had the highest prevalence of Hisayama risk score of 10-year risk > 10%, CACS > 400, and CAD. The high EAVi group (C and D) showed increased risks of CACS > 400 [Group C: adjusted odds ratio, 6.30; 95% confidence interval (CI), 1.39–28.6; Group D: adjusted odds ratio, 9.13; 95% CI, 2.00–41.5] and CAD (Group C: adjusted odds ratio, 2.33; 95% CI, 1.13–4.83; Group D: adjusted odds ratio, 9.13; 95% CI, 2.00–41.5). Multivariate linear regression analysis demonstrated that PCATA_RCA_ was associated with a greater plaque volume independent of EAV index.

**Conclusion:**

Elevated PCAT inflammation is associated with the coronary plaque burden independent of EAV index in patients with CCS.

**Lay summary:**

• This study demonstrates that distinct phenotypes based on ectopic fat volume and composition—the volume of epicardial adipose tissue (EAT) and the inflammation status of pericoronary adipose tissue (PCAT)—can characterize coronary atherosclerotic disease burden in patients with chronic coronary syndrome.

• While both increased EAT volume and PCAT inflammation have been reportedly associated with coronary artery disease (CAD) and cardiovascular events, evidence investigating the association of EAT volume and PCAT inflammation with CAD disease burden is limited.

• Patients with increased EAT volume are at an elevated risk for coronary artery calcification and increased plaque burden, regardless of PCAT inflammation. In contrast, among patients without increased EAT volume, increased PCAT inflammation is correlated with an increased risk of coronary artery calcification and plaque burden.

## Graphical Abstract

**Graphical Abstract:**
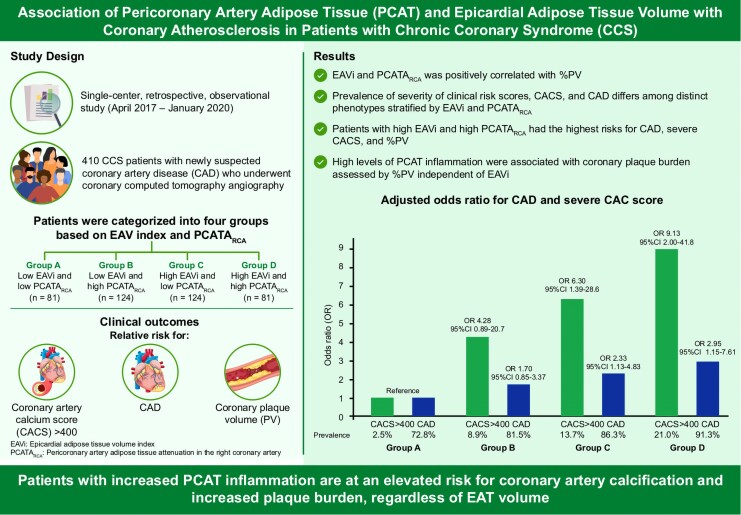
Association of pericoronary artery adipose tissue (PCAT) and epicardial adipose tissue volume with coronary atherosclerosis in patients with chronic coronary syndrome (CCS).

## Introduction

Chronic coronary syndrome (CCS) encompasses a wide clinical spectrum, including both obstructive and non-obstructive coronary artery disease (CAD) that may develop into acute coronary syndrome (ACS) and heart failure.^1^ Recent studies have emphasized that even non-obstructive coronary plaques confer significant risk for future cardiovascular events, highlighting the need for early detection and intervention.^2^ Primary and secondary preventive strategies targeting traditional and novel atherosclerotic risks are key to reducing the atherosclerotic cardiovascular disease (ASCVD) burden.^3,4^ Coronary computed tomography angiography (CCTA) is a first-line diagnostic tool for managing patients with CCS, enabling non-invasive assessment of coronary artery stenosis and plaque burden.^5,6^ In addition to its utility in clinical practice, CCTA has attracted attention for assessing epicardial adipose tissue (EAT) volume and composition.^7–11^ Accumulating evidence has demonstrated that EAT volume (EAV) is associated with heart failure,^11,12^ coronary atherosclerosis,^9,10,13^ and coronary microvascular dysfunction.^9^ These findings suggest a pathophysiological role for EAV in the development of coronary atherosclerosis.

Vascular inflammation from lifestyle-associated diseases promotes atherosclerosis and increases the risk for ASCVD.^3,14^ With the development of imaging technology, CCTA allows for assessing pericoronary adipose tissue (PCAT) inflammation,^15,16^ enabling further risk stratification of cardiovascular death and myocardial infarction.^17,18^ Kuneman *et al*.^19^ demonstrated that PCAT inflammation is increased in patients with ACS, especially in coronary lesions with plaque rupture, where inflammation plays a pivotal role. Although PCAT has been associated with ASCVD events,^17,20^ few studies have examined the relationship between EAV and PCAT inflammation and their association with CAD burden. In this CCTA study, we investigated the associations between distinct phenotypes of PCAT inflammation and EAV with atherosclerotic risk and CAD burden. We tested the hypothesis that high levels of PCAT attenuation (PCATA) are associated with coronary plaque volume independent of EAV and clinical atherosclerotic risk in patients with CCS.

## Methods

### Study population

**Figure 1:**
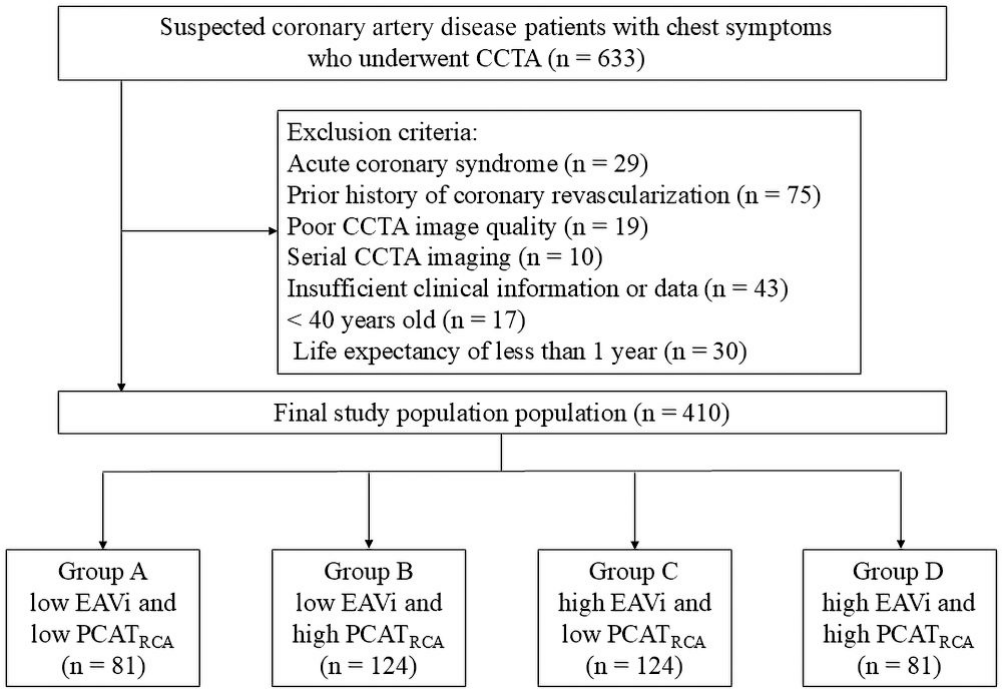
Study flow chart.

**Figure 2:**
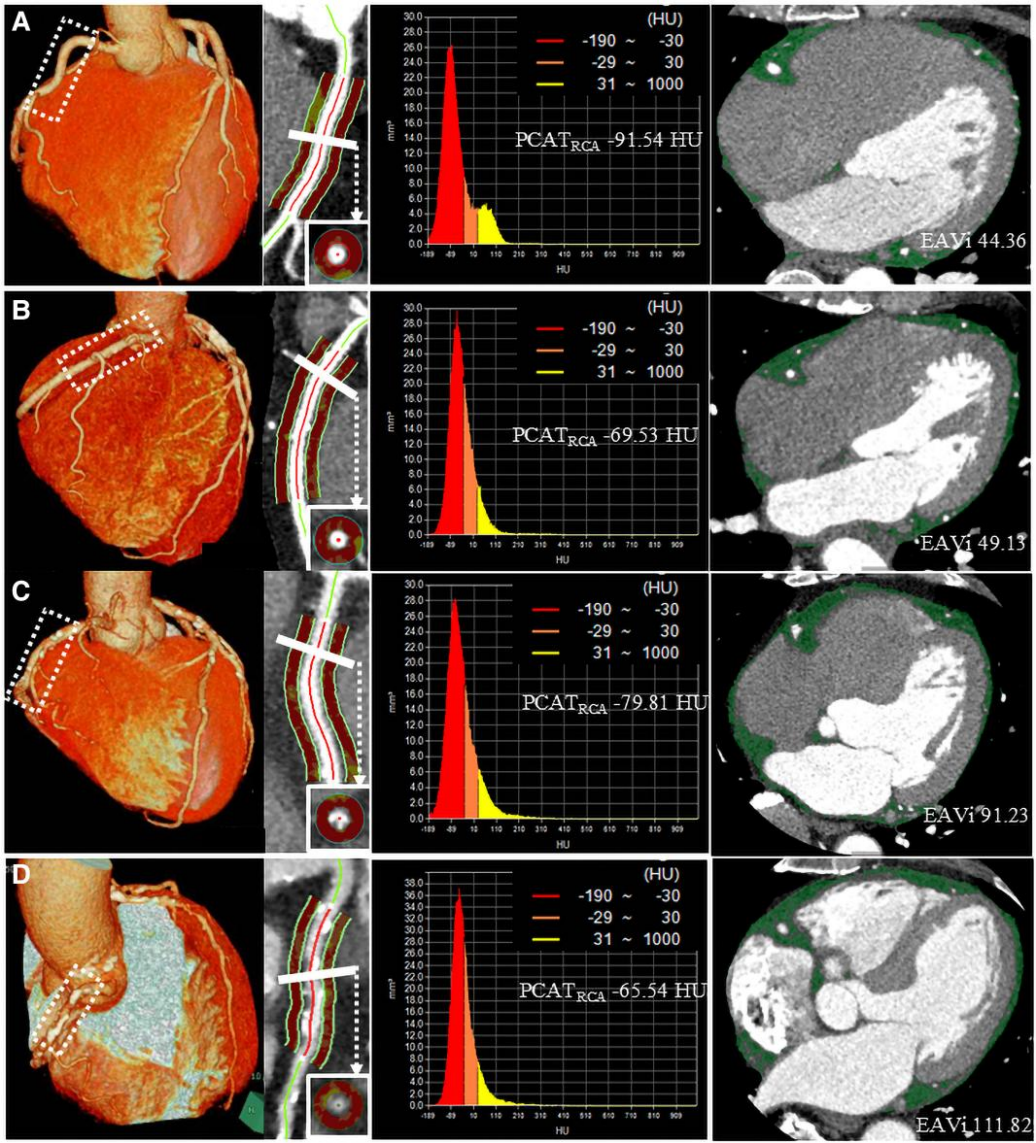
Representative CCTA images of distinct phenotypes and correlation between EAVi and PCATA. (*A*) Low EAVi and low PCAT_RCA_ group, (*B*) low EAVi and high PCAT_RCA_ group, (*C*) high EAVi and low PCAT_RCA_ group, and (*D*) high EAVi and high PCAT_RCA_ group. (*A1–D1*) Volume rendering images (*A2–D2*), pericoronary adipose tissue analysis (*A3–D3*), pericoronary adipose tissue analysis results, and (*A4–D4*) epicardial adipose tissue volume analysis.

This single-centre, retrospective, observational study included patients with newly suspected CAD who underwent CCTA at Kashibaseiki Hospital (Kashiba, Nara, Japan) between April 2017 and January 2020. CCTA was performed based on clinical indications including typical exertional chest pain, atypical chest symptoms, or high-risk coronary profiles in asymptomatic individuals. Patients with CCS without known history of CAD, as defined by the European Society of Cardiology guidelines,^1^ were included in this study. The exclusion criteria were as follows: (i) ACS, (ii) known CAD, (iii) prior coronary revascularization, (iv) poor CCTA image quality, (v) serial CCTA imaging, (vi) insufficient clinical information, (vii) age < 40 years, and (viii) life expectancy of <1 year. Ultimately, 410 CCS patients were included in the analysis (*Figure 1*). To investigate the associations between EAV index (EAVi), PCATA in the right coronary artery (PCATA_RCA_), and CCTA findings, we divided the patients into four groups according to the presence or absence of high EAVi and PCATA_RCA_: Groups A (low EAVi and low PCATA_RCA_), B (low EAVi and high PCATA_RCA_), C (high EAVi and low PCATA_RCA_), and D (high EAVi and high PCATA_RCA_). The median values among the study patients (*n* = 410) were used to classify high and low EAVi and PCATA. EAV was quantified from CCTA images and normalized to body surface area to calculate the EAV index (EAVi), expressed in mL/m^2^ (*Figure 1*).^11^  *Figure 2* shows representative cases for each group.

This study was approved by the ethics committee of Kashibaseiki Hospital (approval no. 2023-A). The requirement for written informed consent was waived by the institutional review board because of the retrospective study design. The Institutional Review Board of Kashibaseiki Hospital approved the pooled data analysis. Additionally, an opt-out process was implemented, allowing patients to refuse or permit the use of their anonymized data. This study was conducted in accordance with the principles of the Declaration of Helsinki.

### Data collection and clinical risk score

Clinical data were collected from the medical records. Laboratory tests were performed within 1 month before CCTA image acquisition. The Hisayama risk score was calculated for all patients to examine the association of EAVi and PCATA_RCA_ with the 10-year ASCVD risk. The Hisayama risk score estimates the absolute 10-year risk of ASCVD, including myocardial infarction and stroke, in the Japanese population.^21^ It consists of sex, systolic blood pressure, abnormal glucose metabolism, serum low density lipoprotein cholesterol, serum high density lipoprotein cholesterol, and smoking. According to the calculated score, a 10-year ASCVD risk of <2% was classified as low risk, 2–10% as moderate risk, and >10% as high risk.

### CCTA acquisition

Image acquisition was performed using a 320-row multidetector CT (Aquilion ONE/NATURE Edition, Canon Medical Systems, Inc., Japan). The scan protocol included non-contrast and contrast-enhanced cardiac CT scan. A non-contrast CT scan measured coronary artery calcium score (CACS).^22^ The abdominal visceral fat area and subcutaneous fat area were assessed at the L2–L3 level in non-contrast-enhanced CT images. Following a non-contrast cardiac CT, CCTA image acquisition was performed with contrast injection at a rate of 2.3–4.9 mL/s. Electrocardiogram (ECG)-synchronized prospective scans were performed within a single breath-hold. Patients were prepared with oral bisoprolol (2.5–5.0 mg) and fast-acting nitrates before CCTA. Scanning parameters were detector collimation of 0.5 × 320 mm, gantry rotation time of 350 ms, tube voltage of 120 kV, and tube current of 130–600 mA. Raw data were reconstructed with optimized algorithms for ECG-synchronized reconstruction and transferred to an offline workstation for further analysis.

### Analysis of CCTA images

CCTA image analysis was performed using SYNAPSE VINCENT version 4.6 (Fujifilm Inc., Tokyo, Japan). The CACS was measured using the Agatston scoring method and classified into the following four categories: 0, 1–100, 101–400, and >400.^23^ CAD severity was categorized into no CAD (no plaque or stenosis), non-obstructive (1–49% stenosis), and obstructive CAD (>50% stenosis).^22^ CAD was defined as non-obstructive and obstructive CAD. Plaque analysis was performed semi-automatically using a TERARECON version 4.4.14. software. The vessel lumen and boundaries of the inner and outer vessels were traced semi-automatically and modified manually, if necessary. The per-patient plaque burden of each major coronary artery was measured and reported as % plaque volume. The per-patient-level plaque burden was calculated as the total PV divided by the total vessel volume.^10^ Plaque components were also categorized as either calcified plaque (CP, >130 HU), or non-calcified plaque (NCP, ≤130 HU), and the coronary plaque burden for each component was calculated.^24^ For patients without CAD, the PV was defined as 0.^6^

### Epicardial and pericoronary artery adipose tissue analysis

EAT was manually delineated on the semi-automatically traced adipose tissue regions using SYNAPSE VINCENT version 4.6 (Fujifilm Inc., Tokyo, Japan). The adipose tissue was defined as the regions with CT attenuation ranging from −190 to −30 Hounsfield units (HU) within the pericardial sac.^8,11^ Image analysis was performed on end-diastolic short- and long-axis slices of contrast-enhanced CT images located from the pulmonary artery trunk bifurcation at the upper slice end towards the apical slice around the ventricle.^25^ EAV was calculated by summing the EAT areas across all CCTA slices. The EAVi, defined as EAV normalized to body surface area, was used for analyses as previously described.

PCATA was semi-automatically assessed using fat attenuation index (FAI) analysis with TERARECON software (version 4.4.14.). It was performed on the three main epicardial coronary arteries. We analysed the proximal 40 mm segments of the left anterior descending (LAD) and left circumflex (LCX) coronary arteries and 10–50 mm proximal to the right coronary artery (RCA).^17^ PCATA was defined as the PCAT with CT attenuation ranging from −190 to −30 HU, located within 3 mm radial distance from the outer vessel walls, as described previously.^18^ The vessel lumen and boundaries of the inner and outer vessel walls were automatically tracked and manually corrected, if necessary. PCATA was reported as the mean CT attenuation value. PCATA_RCA_ was used for the analysis because it is a well-validated standardized model for PCAT analysis and has been associated with cardiovascular events.^15–17^ In cases where the RCA was hypoplastic (<50 mm), PCATA was measured from 10 mm proximal to the greatest extent possible.

### Statistical analysis

Statistical analyses were performed using EZR (Saitama Medical Center, Jichi Medical University, Saitama, Japan), a modified version of the R commander. Continuous variables are presented as mean ± standard deviation or median (interquartile range). Categorical variables are presented as absolute numbers (relative frequencies). For comparisons among groups, continuous variables were analysed using one-way analysis of variance for normally distributed variables and the Kruskal–Wallis test for non-normally distributed variables. The χ^2^ test was used to compare categorical data. Multivariable linear regression analysis was performed to examine the associations of EAVi and PCATA in the LAD, LCX, RCA, and the mean PCATA across the three coronary arteries with % plaque volume. Although PCATA is generally defined as the mean CT attenuation value of PCAT, PCATA_RCA_ was selected for the primary analysis because it represents a well-validated and standardized model for PCAT assessment and has been consistently associated with cardiovascular events.^15–17^ Additionally, we examined the association of EAVi with PCATA_RCA_ using linear regression analysis. Previous studies have indicated that PV is related to factors such as age, sex, lifestyle-related diseases, vascular inflammation, and coronary artery calcification.^6,18^ Therefore, we employed the following adjusted models: Model 1, adjusted for age and sex; Model 2, age, sex, and statin use; Model 3, age, sex, statin use, high Hisayama risk score, high-sensitivity CRP (log-transformed), and CACS (log-transformed); Model 4, age, sex, subcutaneous adipose tissue, and visceral adipose tissue; and Model 5, age, sex, and CCTA indication (patients with typical chest symptoms). Finally, we conducted a logistic regression model to analyse the relative risk of CACS > 400 and CAD (non-obstructive and obstructive) for each group, using Group A (low EAVi and low PCATA_RCA_) as a reference. The model was adjusted for high-risk Hisayama risk scores. To explore interactions between PCATA_RCA_ and EAVi, we conducted interaction analyses using stratified models for % plaque volume and log-transformed CACS. Statistical significance was set at *P* < 0.05 (two-sided).

## Results

### Patient characteristics

**Figure 3:**
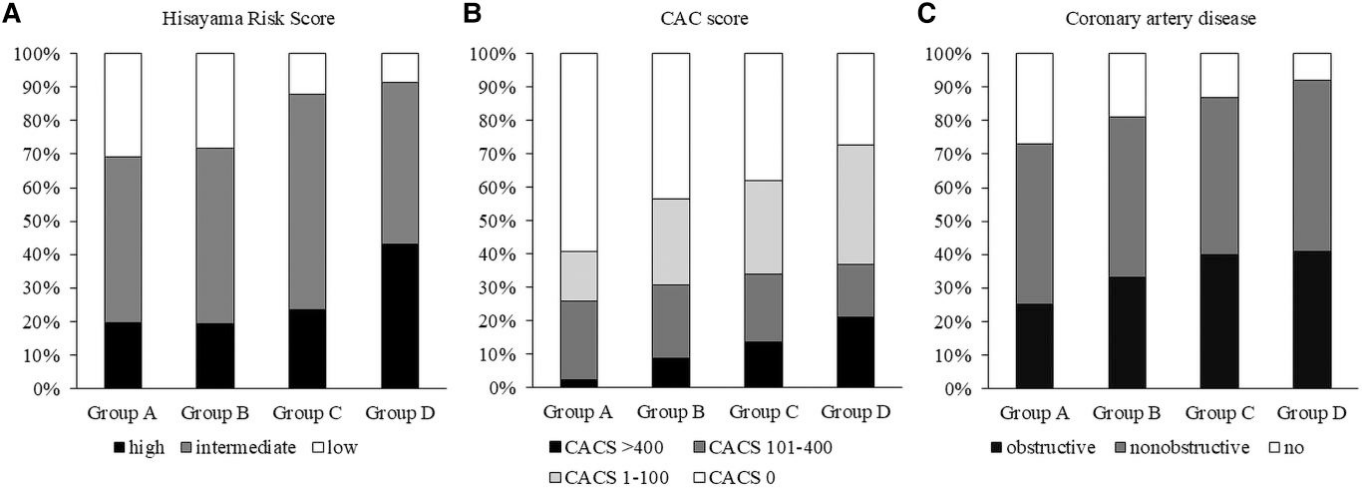
Prevalence of clinical risk score category, severe coronary artery calcium score, and coronary artery disease among the groups. Prevalence of Hisayama risk score category (*A*), coronary artery calcium score (*B*), and severity of coronary artery disease (*C*).

The patient characteristics are summarized in *Table 1*. Briefly, the mean age was 65.7 ± 13.2 years, and 57.6% were male. Hypertension, dyslipidaemia, and diabetes mellitus were present in 73.9%, 72.0%, and 21.7% of patients, respectively. Patients were categorized into four groups based on the median values of PCATA_RCA_ (−76.6 HU) and EAVi (73.5 mL/mm²). A higher proportion of males was observed in Groups B, and D (with high PCATA_RCA_), and Group D had the greatest percentage of patients with high Hisayama risk scores (43.2%, *P* < 0.001; *Figure 3A*). Hypertension and diabetes were more prevalent in Group D, while serum high-sensitivity CRP levels were elevated in Groups C and D.

**Table 1:** Patient characteristics and CCTA findings

	Overall (*n* = 410)	Group A Low EATVi Low PCATA (*n* = 81)	Group B Low EATVi High PCATA (*n* = 124)	Group C High EATVi Low PCATA (*n* = 124)	Group D High EATVi High PCATA (*n* = 81)	*P*-value
Baseline patient characteristics						
Age, years	65.7 ± 13.2	61.6 ± 13.2	62.0 ± 13.6	68.0 ± 11.7	72.1 ± 11.7	<0.001
Male	236 (57.6%)	42 (51.9%)	81 (65.3%)	60 (48.4%)	53 (65.4%)	0.015
Body mass index, kg/m^2^	24.5 ± 3.8	23.2 ± 4.0	23.7 ± 3.5	25.4 ± 3.6	25.4 ± 3.5	<0.001
Current smoker	63 (15.4%)	17 (21.0%)	19 (15.3%)	18 (14.5%)	9 (11.1%)	0.367
Hypertension	303 (73.9%)	48 (59.3%)	90 (72.6%)	95 (76.6%)	70 (86.4%)	0.001
Dyslipidaemia	295 (72.0%)	61 (75.3%)	82 (66.1%)	97 (78.2%)	55 (67.9%)	0.132
Diabetes mellitus	89 (21.7%)	12 (14.8%)	19 (15.3%)	33 (26.6%)	25 (30.9%)	0.012
Beta-blockers	27 (6.6%)	4 (4.9%)	10 (8.1%)	7 (5.6%)	6 (7.4%)	0.785
RAS-inhibitor	95 (23.2%)	13 (16.0%)	23 (18.5%)	29 (23.4%)	30 (37.0%)	0.006
Calcium channel blockers	113 (27.6%)	19 (23.5%)	31 (25.0%)	32 (25.8%)	31 (38.3%)	0.115
Statin	101 (24.6%)	15 (18.5%)	28 (22.6%)	34 (27.4%)	24 (29.6%)	0.318
LDL cholesterol, mg/dL	123.2 ± 37.1	125.5 ± 36.8	118.6 ± 34.5	128.6 ± 40.5	120.4 ± 36.1	0.239
HDL cholesterol, mg/dL	63.7 ± 18.4	71.1 ± 21.3	65.4 ± 17.3	59.0 ± 16.7	60.3 ± 16.5	<0.001
Triglyceride, mg/dL	126 (89–172)	103 (78–160)	119 (80–166)	139 (97–185)	141 (103–176)	0.016^a^
High-sensitive CRP, mg/L	0.9 (0.4–2.3)	0.6 (0.3–1.2)	0.7 (0.3–2.0)	1.1 (0.5–2.5)	1.1 (0.6–3.9)	<0.001^a^
Haemoglobin A1c, %	6.1 ± 1.1	5.8 ± 0.5	5.8 ± 0.9	6.4 ± 1.6	6.2 ± 0.9	<0.001
Hisayama risk score						
High risk	104 (25.4%)	16 (19.8%)	24 (19.4%)	29 (23.4%)	35 (43.2%)	<0.001
Intermediate risk	224 (54.6%)	40 (49.4%)	65 (52.4%)	80 (64.5%)	39 (48.1%)	0.060
Low risk	82 (20.0%)	25 (30.9%)	35 (28.2%)	15 (12.1%)	7 (8.6%)	<0.001
CCTA findings						
CAD	341 (83.2%)	59 (72.8%)	101 (81.5%)	107 (86.3%)	74 (91.3%)	0.011
Non-obstructive CAD	198 (48.3%)	39 (48.1%)	60 (48.4%)	58 (46.8%)	41 (50.6%)	0.962
Obstructive CAD	143 (34.9%)	20 (24.7%)	41 (33.1%)	49 (39.5%)	33 (40.7%)	0.099
CACS, Agatston unit^a^	17.7 (0–174.3)	0 (0–104.0)	9.3 (0–162.5)	25.6 (0–186.0)	57.6 (0–314.5)	0.001
CACS 0	171 (41.7%)	48 (59.3%)	54 (43.5%)	47 (37.9%)	22 (27.2%)	<0.001
CACS 1–100	108 (26.3%)	12 (14.8%)	32 (25.8%)	35 (28.2%)	29 (35.8%)	0.023
CACS 101–400	84 (20.5%)	19 (23.5%)	27 (21.8%)	25 (20.2%)	13 (16.0%)	0.670
CAC > 100	131 (32.0%)	21 (25.9%)	38 (30.6%)	42 (33.9%)	30 (37.0%)	0.453
CACS > 400	47 (11.5%)	2 (2.5%)	11 (8.9%)	17 (13.7%)	17 (21.0%)	0.002
Subcutaneous adipose tissue area, cm^2^	157.4 ± 76.8	149.2 ± 82.4	138.0 ± 80.2	178.4 ± 65.1	163.1 ± 75.1	<0.001
Visceral adipose tissue area, cm^2^	107.4 ± 57.1	82.3 ± 44.1	84.0 ± 47.8	132.2 ± 59.6	130.9 ± 52.8	<0.001
% total plaque volume, %	36.3 ± 19.8	30.5 ± 21.4	37.2 ± 20.2	37.5 ± 18.3	38.9 ± 19.2	0.027
% calcified plaque volume, %	12.9 ± 7.7	10.6 ± 7.9	13.0 ± 7.7	13.5 ± 7.3	14.3 ± 8.1	0.016
% non-calcified plaque volume, %	23.4 ± 12.7	19.9 ± 13.9	24.2 ± 13.0	24.0 ± 11.9	24.6 ± 11.9	0.058

Variables were expressed as *n* (%) or mean (SD). Low PCAT FAI was defined as <−76.6 HU, and high PCAT FAI was defined as ≥−76.6 HU. Low EAVi was defined as <73.5 mL/mm^2^, and high EAVi was defined as ≥73.5 mL/mm^2^.

CACS, coronary artery calcium score; CAD, coronary artery disease; CCTA, coronary computed tomography angiography; CRP, C-reactive protein; EAVi, epicardial adipose tissue volume index; HDL, high density lipoprotein; LDL, low density lipoprotein; PCATA, pericoronary adipose tissue attenuation; RAS, renin-angiotensin system; RCA, right coronary artery.

^a^Analyses using log-transformed.

### CCTA findings

Regarding CAD severity, 48.3% (*n* = 198) had non-obstructive CAD, and 34.9% (*n* = 143) had obstructive CAD, totalling 83.2% (*n* = 342) of patients (*Table 1*). The median CACS was 17.7 (interquartile range: 0–174.3), and the mean EAVi was 75.7 ± 28.3 mL/mm^2^. The prevalence of CAC > 400 was 11% (47 patients) in the overall cohort. The mean PCATA value for each coronary artery was −76.8 ± 9.2 HU in RCA, −76.7 ± 7.3 HU in LAD, and −73.5 ± 6.9 HU in LCX.

*Table 1* summarizes the CCTA findings across the four groups. The log-transformed coronary artery calcium score [log(CAC + 1)] increased progressively, with Group D showing the highest values (*Table 1*). While the proportion of patients with CAC > 100 did not differ significantly among the groups, Group D exhibited a significantly greater prevalence of CAC > 400 (Group A, 2.5%; Group B, 8.9%; Group C, 13.7%; Group D, 21.0%; *P* = 0.002), indicating more advanced coronary calcification. This pattern is further illustrated in *Figure 3B*. In parallel, the prevalence of CAD was also highest in Group D (Group A, 72.8%; Group B, 81.5%; Group C, 86.3%; Group D, 91.3%; *P* = 0.011; *Figure 3C*). Additionally, Group D, characterized by both high PCATA_RCA_ and high EAVi, showed the greatest total plaque burden (Group A, 30.4%; Group B, 37.2%; Group C, 37.5%; Group D, 38.9%; *P* = 0.027).

### Relative risk for CAD and severe CACS

We evaluated the relative risk of severe coronary calcification (CACS > 400) and CAD according to EAVi and PCATA_RCA_ categories. Multivariable logistic regression analysis, adjusted for the Hisayama risk score (>10% estimated 10-year ASCVD risk), was performed to analyse the relative risk of CACS > 400 and CAD in each group compared with Group A (*Graphical Abstract*). Patients with high EAVi (Groups C and D) demonstrated significantly elevated risk for CACS > 400 (Group C: adjusted OR, 6.30; 95% CI, 1.39–28.6; *P* = 0.017; Group D: adjusted OR, 9.13; 95% CI, 2.00–41.5; *P* = 0.004) and CAD (Group C: adjusted OR, 2.33; 95% CI, 1.13–4.83; *P* = 0.023; Group D: adjusted OR, 9.13; 95% CI, 2.00–41.5; *P* = 0.025). Patients with low EAVi but high PCATA_RCA_ (Group B) exhibited a numerically higher risk for CACS > 400 (adjusted OR, 4.28; 95% CI, 0.89–20.7; *P* = 0.071) and CAD (adjusted OR, 1.70, 95% CI, 0.85–2.27; *P* = 0.132), although these associations did not reach statistical significance.

Additional analyses separately evaluated the relative risks of obstructive and non-obstructive CAD according to EAVi and PCATA_RCA_ categories (see Supplementary data online, *Figure S1*). No statistically significant differences in the relative risks of non-obstructive and obstructive CAD were observed among the groups. However, a trend towards a higher % plaque volume was noted in the obstructive CAD group. Collectively, these findings suggest that a high EAVi, particularly when combined with elevated PCATA_RCA_, is associated with a substantially increased risk of severe coronary calcification and the presence of CAD in patients with CCS.

### Factors associated with pericoronary adipose tissue

Supplementary data online, *Table S3* presents the results of linear regression analysis for PCATA_RCA_. The factors associated with PCATA_RCA_ included male sex (β coefficient = 4.333, *P* < 0.001), hypertension (β coefficient = 3.065, *P* = 0.003), dyslipidaemia (β coefficient = −2.576, *P* = 0.011), EAVi (β coefficient = −0.092, *P* < 0.001), subcutaneous adipose tissue area (β coefficient = −0.019, *P* < 0.001), visceral adipose tissue area (β coefficient = −0.019, *P* = 0.020), and %total plaque volume (β coefficient = 0.054, *P* = 0.019). There was no significant correlation with CRP, CACS, and CAD severity.

### Factors associated with coronary atherosclerosis

We evaluated the associations of EAVi and PCATA_RCA_ with % total plaque volume using univariate and multivariable linear regression analyses (*Table 2*). In the unadjusted model, both EAVi (β = 0.115; 95% CI, 0.048–0.183; *P* < 0.001) and PCATA_RCA_ (β = 0.251; 95% CI, 0.042–0.460; *P* = 0.019) were significantly associated with % plaque volume. In multivariable models, PCATA_RCA_ consistently showed a significant association with % plaque volume independent of clinical and imaging variables, while the association of EAVi with % plaque volume was attenuated after adjustment. Specifically, PCATA_RCA_ remained significantly associated with plaque volume after adjustment for age and sex (Model 1: β = 0.252; 95% CI, 0.041–0.462; *P* = 0.019), and after further adjustment for statin use (Model 2: β = 0.248; 95% CI, 0.038–0.459; *P* = 0.021). The association persisted even after additional adjustment for high Hisayama risk score, hs-CRP, and CACS (Model 3: β = 0.225; 95% CI, 0.018–0.431; *P* = 0.033). Similarly, PCATA_RCA_ remained significantly associated with plaque volume in models adjusting for age, sex, subcutaneous and visceral adipose tissue areas (Model 4: β = 0.250; 95% CI, 0.037–0.462; *P* = 0.021) and for age, sex, and CCTA indication (Model 5: β = 0.277; 95% CI, 0.065–0.489; *P* = 0.010). In contrast, the association between EAVi and plaque volume was not statistically significant after multivariable adjustment. These findings suggest that PCATA_RCA_ is a robust and independent marker of coronary plaque burden, even after accounting for traditional atherosclerotic risk factors and body fat distribution.

**Table 2:** Factors associated with % plaque volume in multivariable linear regression models

		β coefficient	95% CI	*P*-value
Unadjusted model	EAVi	0.115	0.048–0.183	<0.001
	PCATA_RCA_	0.251	0.042–0.460	0.019
Model 1	EAVi	0.065	−0.006–0136	0.073
	PCATA_RCA_	0.252	0.041–0.462	0.019
Model 2	EAVi	0.062	−0.010–0.134	0.092
	PCATA_RCA_	0.248	0.038–0.459	0.021
Model 3	EAVi	0.057	−0.014–0.128	0.116
	PCATA_RCA_	0.225	0.018–0.431	0.033
Model 4	EAVi	0.069	−0.019–0.158	0.123
	PCATA_RCA_	0.250	0.037–0.462	0.021
Model 5	EAVi	0.065	−0.006–0.136	0.073
	PCATA_RCA_	0.277	0.065–0.489	0.010

Abbreviations as in *Table 1*. Model 1: adjusted by age and sex. Model 2: adjusted by age, sex, and statin use. Model 3: adjusted by age, sex, statin use, high Hisayama risk score, high-sensitive CRP (log-transformed), and CACS (log-transformed). Model 4: adjusted by age, sex, subcutaneous adipose tissue, and visceral adipose tissue. Model 5: adjusted by age, sex, and CCTA indication (patients with typical chest symptoms).

Similarly, we examined the associations between PCATA in the LAD, LCX, and the mean PCATA across the three coronary arteries with % plaque volume. However, no significant association was observed between PCATA in the LAD or LCX and % plaque volume (see Supplementary data online, *Table S1*). In addition, logistic regression analysis was performed to evaluate the independent associations of EAVi and PCATA_RCA_ with %NCP and %CP. In the multivariable-adjusted models, both %NCP and %CP were significantly associated with PCATA_RCA_, independent of EAVi. These findings are described in detail in the Supplementary Materials (see Supplementary data online, *Table S2*).

### Interaction between epicardial and pericoronary adipose tissue in relation to plaque burden and calcification

Next, we conducted interaction analyses to explore potential interactions, for % plaque volume, between PCATA_RCA_ and EAVi status (high vs. low) or between EAVi and PCATA status (high vs. low). The corresponding *P*-values were 0.185 and 0.224, respectively (see Supplementary data online, *Table S4*). Similarly, for log-transformed CACS, the interaction *P*-values were 0.215 and 0.291, respectively. It suggested independent contributions of PCATA_RCA_ and EAVi to plaque burden and CACS. In addition, interactions for % plaque volume were tested between EAV and EAVi status (high vs. low) or between EAVi and EAV status (high vs. low). The corresponding *P*-values were 0.183 and 0.121, respectively (see Supplementary data online, *Table S4*). Similarly, for log-transformed CACS, the interaction *P*-values were 0.501 and 0.797, respectively (see Supplementary data online, *Table S4*). It suggested that no interaction was observed between EAV and EAVi.

## Discussion

In this study of patients with CCS undergoing CCTA, we comprehensively evaluated the associations of EAVi and PCATA with coronary atherosclerotic burden and clinical risk. The main findings are summarized as follows: (i) EAVi and PCATA_RCA_ were positively correlated with % plaque volume for the overall population; (ii) CACS, plaque burden, and prevalence of CAD varied significantly among patient phenotypes stratified by EAVi and PCATA_RCA_; (iii) patients with high EAVi and PCATA_RCA_ had the highest risk for CAD, severe CACS, and % plaque volume; and (iv) high levels of PCAT inflammation were associated with coronary plaque burden independent of EAVi. Our findings provide insights into the heterogeneity of EAV and the inflammatory status of PCAT, serving as an imaging marker of coronary atherosclerosis in patients with CCS.

### EAT and PCAT as markers for coronary atherosclerosis

EAT, an ectopic fat, plays a pivotal role in the pathogenesis of coronary atherosclerosis through distinctive transcriptomes, unlike other fats such as visceral and subcutaneous fat.^26^ Although CACS is a marker of increased disease burden in CAD, increased EAT has been associated with obstructive CAD independent of CACS.^27,28^ Additionally, previous studies have demonstrated that increased EAT independently predicts obstructive CAD and high-risk plaques.^7,10^ Recently, PCAT FAI has been shown to correlate with PCAT inflammation^15^ and cardiovascular events.^17^ These findings demonstrate the clinical utility of PCAT as a marker of local inflammation for risk stratification of patients with CCS.^16^ However, studies investigating the association between the EAT burden and PCAT are limited.

In this study, we demonstrated an inverse correlation between EAVi and PCATA_RCA_ in the overall population. Adipose tissue remodelling is prominent in the visceral adipose tissue and comprises distinct types of immune cells, angiogenesis, and extracellular matrix accumulation.^9,13,29^ The radiographic signature of fat density can be influenced by the hypertrophy and fibrosis of adipocytes, which determine the CT signal attenuation around the coronary arteries. Although hypertrophic fat deposits exhibit low density,^30^ increased EAT density can be influenced by inflammation and fibrosis, which mitigates the expected effect of hypertrophic fat cells on fat CT signal attenuation.^31^ Despite the limited ability of CCTA to assess PCAT inflammation and fibrosis, we demonstrated that the high EAVi and PCAT_RCA_ groups had the highest CAD risk. This finding indicates a synergistic effect in assessing the volume and composition of ectopic fat. In multivariable analyses, however, PCATA_RCA_ consistently showed stronger and more robust associations with plaque burden across multiple models. The independent association between PCAT inflammation and coronary plaque burden facilitates the assessment of PCAT inflammation as a driving marker of coronary atherosclerosis and a primary preventive strategy in patients with CCS.

Similarly, the average PCATA across the three main coronary arteries also showed a significant association with % total plaque volume independent of EAVi in the multivariable-adjusted model. However, no significant associations were observed for PCATA measured individually in the LAD or LCX. One possible explanation is that the RCA is surrounded by a greater volume of perivascular fat and has fewer side branches, potentially leading to more stable measurements and fewer imaging artefacts compared with the LAD or LCX.^17^ These anatomical differences may contribute to the more robust association observed between PCATA_RCA_ and coronary plaque burden.

### PCAT as a therapeutic target

Invasive therapy for myocardial ischaemia is a standard and effective management strategy for CCS.^32^ Medical therapy targeting lifestyle-associated diseases such as obesity, hypertension, dyslipidaemia, and diabetes mellitus is crucial for preventing ASCVD events in patients with CCS.^33^ Moreover, recent clinical studies have shown that targeting inflammation in patients at high risk for ASCVD is effective.^3,33^ The CANTOS trial demonstrated that vascular inflammation treatments reduce the risk of ASCVD.^34^ Additionally, several trials have shown that colchicine, an oral anti-inflammatory drug, reduces the risk of cardiovascular events.^35^ Therefore, biomarkers identifying vascular inflammation are important for further risk stratification of patients with CCS and may serve as therapeutic targets for anti-inflammatory drugs.

In addition to the risk stratification of patients with CCS, treatments targeting adipose tissue as a residual risk marker have been considered. Statins are among the most important standard treatments for CAD. In a sub-analysis of postmenopausal women using statins, the high-dose atorvastatin group showed a reduction in EAT volume and FAI values after 1 year.^36^ In our study, PCATA_RCA_ was independently associated with coronary plaque burden even after adjusting for atherosclerotic risks, including statin therapy. Additionally, SGLT2 inhibitors, GLP-1 receptor agonists, and anti-inflammatory drugs have been reported to reduce EAT volume.^1,4,9^

In this study, we used the median values of PCATA_RCA_ as cut-off points, providing an increased risk of CACS > 400 and CAD compared with those with low EAVi and PCATA_RCA_. Oikonomou *et al*. demonstrated that PCATA > −70 HU was associated with poorer cardiovascular outcomes,^17,18^ which technically cannot be applied to other studies. Instead, the 50th percentile of the PCATA measured in the RCA or LAD was shown to be associated with a two-fold increased risk of predicting cardiovascular events.^15,16^ This finding suggests that PCATA above the 50th percentile can be a marker of pericoronary inflammation to predict CAD and cardiovascular events in patients with CCS. Further studies are required to investigate the effects of these pharmacological therapies on PCAT inflammation and the disease burden of coronary atherosclerosis.

### Limitations

Our study has the following limitations. First, we excluded CCS patients with known CAD and prior coronary revascularization and did not assess patients with vasospastic angina or microvascular dysfunction, in whom PCAT and EAT may contribute to coronary microvascular dysfunction.^37^ Secondly, since this study included only patients with CCS, our observations cannot be extrapolated to patients with ACS, who have been shown to have higher PCATA scores than those with CCS.^19^ Thirdly, although we adjusted for multiple major clinical and imaging variables, the potential for residual confounding cannot be excluded. Specifically, we were unable to account for certain factors such as inflammatory biomarkers, physical activity levels, and other lifestyle-related variables, which may have influenced the associations observed. Fourthly, because the Hisayama risk score was specifically developed for the Japanese population, the generalizability of our risk assessment to other ethnic groups may be limited. However, a high Hisayama risk score is defined as a 10-year ASCVD risk > 10%, which aligns with thresholds used in other international risk models. Finally, further studies are needed to investigate the prognostic implications of EAVi and PCATA_RCA_ on cardiovascular outcomes in association with responses to pharmacological medical therapy and lifestyle modifications.

## Conclusion

The epicardial and pericoronary adipose tissue burden and composition vary among patients with CCS who undergo CCTA. Our results demonstrate that high levels of PCATA_RCA_ are drivers of coronary atherosclerosis, independent of EAV. Further studies are needed to investigate the clinical implications of EAVi and PCATA_RCA_ as risk stratification markers and therapeutic targets.

## Supplementary Material

qyaf079_Supplementary_Data

## Data Availability

The participants of this study did not give written consent for their data to be shared publicly, so due to the sensitive nature of the research, supporting data are not available.

## Lead author biography

Affiliation: PhD graduate student, Department of Cardiovascular Medicine, Osaka Metropolitan University Graduate School of Medicine. Qualifications: Medical Doctor, Osaka City University School of Medicine in 2018; Board Certified Doctor of Internal Medicine; Fellow of the Japanese Association of Cardiovascular Intervention and Therapeutics. Honours: YIA of the Japanese Society of Cardiovascular Imaging & Dynamics Congress 2024. Key papers: as a first author: Yamaura H, *et al*. Front Cardiovasc Med. 2022;9:824470. PMID: 35463764; as a co-author: Ishikawa H, *et al*. Eur Heart J Imaging Methods Pract. 2024;2(1):qyae056. PMID: 39224094.
