# Supplementary material for: High levels of pericoronary adipose tissue inflammation are associated with coronary atherosclerosis independent of epicardial adipose tissue volume in patients with chronic coronary syndrome
Source: Eur Heart J Imaging Methods Pract. 2025 Jun 10;3(2):qyaf079. doi: 10.1093/ehjimp/qyaf079 (PMC12238945; doi:10.1093/ehjimp/qyaf079)

**Supplementary materials**

**High levels of pericoronary adipose tissue inflammation are associated with coronary atherosclerosis independent of epicardial adipose tissue volume in patients with chronic coronary syndrome**

Hiroki Yamaura, MD^a^; Kenichiro Otsuka, MD, PhD^a^; Hirotoshi Ishikawa, MD, PhD^b^; Kana Hojo, MD^a^; Kotaro Matsumoto, MD^a^; Naoki Fujisawa, MD^a^; Akihiro Okamoto, MD^a^; Tomohiro Yamaguchi, MD, PhD^a^; Shunsuke Kagawa, MD^a^; Takenobu Shimada, MD^a^; Atsushi Shibata, MD, PhD^a^; Asahiro Ito, MD, PhD^a^; Takanori Yamazaki, MD, PhD^a^; Kenei Shimada, MD, PhD^b^; Noriaki Kasayuki, MD, PhD^b^; Daiju Fukuda, MD, PhD^a^

^a^Department of Cardiovascular Medicine, Osaka Metropolitan University Graduate School of Medicine, Osaka, Japan

^b^Department of Cardiovascular Medicine, Kashibaseiki Hospital, Kashiba, Japan

| **Table S1. Factors associated with %plaque volume in multivariable linear regression models.** | | | | |
| --- | --- | --- | --- | --- |
|  |  | **β Coefficient** | **95%CI** | **p-value** |
|  | **PCATA in LAD** | | | |
| **Unadjusted model** | EAVi | 0.115 | 0.048–0.183 | <0.001 |
|  | PCATA_LAD_ | 0.148 | -0.117–0.412 | 0.273 |
| **Model 1** | EAVi | 0.058 | -0.018–0.133 | 0.134 |
|  | PCATA_LAD_ | 0.191 | -0.088–0.470 | 0.179 |
| **Model 2** | EAVi | 0.054 | -0.022–0.130 | 0.162 |
|  | PCATA_LAD_ | 0.192 | -0.086–0.471 | 0.175 |
| **Model 3** | EAVi | 0.053 | -0.022–0.128 | 0.163 |
|  | PCATA_LAD_ | 0.184 | -0.087–0.455 | 0.182 |
|  | **PCATA in LCX** | | | |
| **Unadjusted model** | EAVi | 0.115 | 0.048–0.183 | <0.001 |
|  | PCATA_LCX_ | 0.186 | -0.091–0.463 | 0.189 |
| **Model 1** | EAVi | 0.055 | -0.017–0.456 | 0.133 |
|  | PCATA_LCX_ | 0.169 | -0.119–0.456 | 0.249 |
| **Model 2** | EAVi | 0.051 | -0.021–0.123 | 0.164 |
|  | PCATA_LCX_ | 0.167 | -0.120–0.455 | 0.254 |
| **Model 3** | EAVi | 0.044 | -0.026–0.115 | 0.217 |
|  | PCATA_LCX_ | 0.128 | -0.151–0.407 | 0.369 |
|  | **mean PCATA across three coronary arteries** | | | |
| **Unadjusted model** | EAVi | 0.115 | 0.048–0.183 | <0.001 |
|  | PCATA_mean_ | 0.251 | 0.042–0.460 | 0.019 |
| **Model 1** | EAVi | 0.068 | 0.006–0.142 | 0.071 |
|  | PCATA_mean_ | 0.325 | 0.023–0.627 | 0.035 |
| **Model 2** | EAVi | 0.065 | -0.009–0.139 | 0.086 |
|  | PCATA_mean_ | 0.324 | 0.022–0.626 | 0.036 |
| **Model 3** | EAVi | 0.061 | -0.013–0.134 | 0.104 |
|  | PCATA_mean_ | 0.300 | 0.005–0.595 | 0.046 |
| Abbreviations as in Table 1.  Model 1: Adjusted by age and sex  Model 2: Adjusted by age, sex, and statin use  Model 3: Adjusted by age, sex, statin use, high Hisayama risk score, high-sensitive CRP (log-transformed), and CACS (log-transformed)  Model 4: Adjusted by age, sex, statin use, and CCTA indication (typical chest symptom) | | | | |

| **Table S2. Univariate and multivariable linear regression models for associations of EAVi and PCATA_RCA_ with calcified and non-calcified coronary plaque volume** | | | | |
| --- | --- | --- | --- | --- |
|  |  | **β Coefficient** | **95%CI** | **p-value** |
|  | **Calcified plaque volume** | | | |
| **Unadjusted model** | EAVi | 0.050 | 0.024–0.077 | <0.001 |
|  | PCATA_RCA_ | 0.095 | 0.013–0.176 | 0.023 |
| **Model 1** | EAVi | 0.026 | -0.001–0.054 | 0.059 |
|  | PCATA_RCA_ | 0.095 | 0.014–0.176 | 0.021 |
| **Model 2** | EAVi | 0.026 | -0.002–0.053 | 0.068 |
|  | PCATA_RCA_ | 0.094 | 0.014–0.175 | 0.022 |
| **Model 3** | EAVi | 0.021 | -0.005–0.048 | 0.119 |
|  | PCATA_RCA_ | 0.079 | 0.002–0.156 | 0.045 |
| **Model 4** | EAVi | 0.026 | -0.008–0.060 | 0.134 |
|  | PCATA_RCA_ | 0.093 | 0.012–0.175 | 0.025 |
|  |  | **β Coefficient** | **95%CI** | **p-value** |
|  | **Non-calcified plaque volume** | | | |
| **Unadjusted model** | EAVi | 0.062 | 0.018–0.106 | 0.005 |
|  | PCATA_RCA_ | 0.150 | 0.016–0.284 | 0.028 |
| **Model 1** | EAVi | 0.037 | -0.010–0.084 | 0.125 |
|  | PCATA_RCA_ | 0.150 | 0.012–0.288 | 0.033 |
| **Model 2** | EAVi | 0.034 | -0.013–0.081 | 0.159 |
|  | PCATA_RCA_ | 0.147 | 0.009–0.285 | 0.036 |
| **Model 3** | EAVi | 0.034 | -0.014–0.081 | 0.161 |
|  | PCATA_RCA_ | 0.141 | 0.003–0.278 | 0.045 |
| **Model 4** | EAVi | -0.002 | -0.023–0.020 | 0.859 |
|  | PCATA_RCA_ | 0.150 | 0.011–0.029 | 0.035 |
| Abbreviations as in Table 1  Model 1: Adjusted by age and sex  Model 2: Adjusted by age, sex, and statin use  Model 3: Adjusted by age, sex, statin use, high Hisayama risk score, high-sensitive CRP (log-transformed), and CACS (log-transformed)  Model 4: Adjusted by age, sex, subcutaneous adipose tissue and visceral adipose tissue | | | | |

| **Table S3. Linear regression model for factors associated with pericoronary adipose tissue attenuation in right coronary artery.** | | | |
| --- | --- | --- | --- |
|  | Unadjusted model | | |
|  | β Coefficient | 95% CI | p value |
| Age, years | -0.011 | -0.079 - 0.057 | 0.758 |
| Male | 4.333 | 2.570 – 6.100 | <0.001 |
| Body mass index, kg/m^2^ | 0.023 | -0.215 – 0.260 | 0.852 |
| Current smoker | 0.991 | -1.504 – 3.487 | 0.435 |
| Hypertension | 3.065 | 1.043 – 5.088 | 0.003 |
| Dyslipidemia | -2.576 | -4.558 - -0.593 | 0.011 |
| Diabetes mellitus | -0.328 | -2.517 – 1.861 | 0.768 |
| CKD | 0.553 | -1.467 – 2.573 | 0.591 |
| Hisayama Risk Score, high risk | 1.880 | -0.183 – 3.942 | 0.074 |
| CRP, mg/L * | 0.642 | -0.010 – 1.294 | 0.053 |
| LDL-Cholesterol, mg/dL | -0.035 | -0.061 - -0.008 | 0.012 |
| HDL-Cholesterol, mg/dL | -0.024 | -0.079 – 0.031 | 0.391 |
| Triglyceride, mg/dL * | 0.000 | -0.004 – 0.004 | 0.962 |
| Hemoglobin A1c, % | -0.657 | -1.680 – 0.365 | 0.207 |
| EATVi, mL/m^2^ | -0.092 | -0.122 - -0.061 | <0.001 |
| Subcutaneous adipose tissue area, cm^2^ | -0.019 | -0.030 - -0.007 | 0.001 |
| Visceral adipose tissue area, cm^2^ | -0.019 | -0.034 - -0.003 | 0.020 |
| CACS, Agatston unit * | 0.229 | -0.110 – 0.568 | 0.185 |
| obstructive CAD | 0.206 | -1.680 – 2.092 | 0.830 |
| CAD | 1.667 | -0.720 – 4.053 | 0.170 |
| % total plaque volume | 0.251 | 0.042 – 0.460 | 0.019 |
| % calcified plaque volume | 0.150 | 0.016 – 0.284 | 0.028 |
| % non-calcified plaque volume | 0.095 | 0.013 – 0.176 | 0.023 |
| CACS = coronary artery calcium score, CKD = chronic kidney disease, CRP = C-reactive protein, EAVi = epicardial adipose tissue volume index, HDL = high-dense lipoprotein, LDL = low-dense lipoprotein, PCATA = pericoronary adipose tissue attenuation, RCA = right coronary artery. | | | |

| **Table S4. Interaction analysis between epicardial and pericoronary adipose tissue in relation to plaque burden and calcification.** | | |
| --- | --- | --- |
|  | interaction p value | |
|  | % plaque volume | log [CAC+1] |
| PCATA_RCA_ × EAVi status (high vs low) | 0.185 | 0.215 |
| EAVi × PCATA_RCA_ status (high vs low) | 0.224 | 0.291 |
| EAV × EAVi status (high vs low) | 0.183 | 0.501 |
| EAVi × EAV status (high vs low) | 0.121 | 0.797 |
| CACS = coronary artery calcium score, EAV = epicardial adipose tissue volume, EAVi = epicardial adipose tissue volume index, PCATA = pericoronary adipose tissue attenuation, RCA = right coronary artery. | | |

**Supplementary Material, Figure S1**

Additional subgroup analyses were performed to separately evaluate the odds ratios for obstructive and non-obstructive coronary artery disease (CAD) across the four study groups defined by EAVi and PCATA_RCA_ status. No statistically significant differences in the odds ratios were observed between groups for either CAD subtype. However, a trend toward higher % total plaque volume and increased prevalence of obstructive CAD was observed in Group D (high EAVi and high PCATA_RCA_). EAVi, epicardial adipose tissue volume index; PCATA, pericoronary adipose tissue attenuation; RCA, right coronary artery


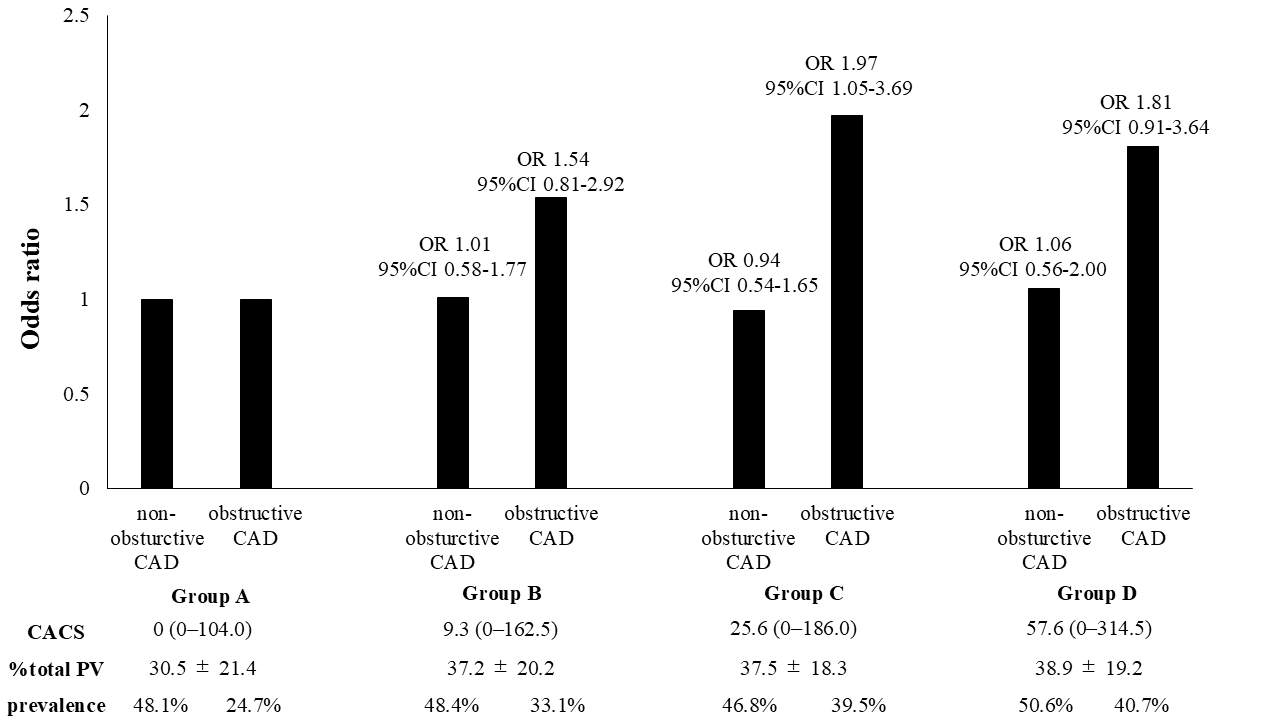

Supplement: qyaf079_Supplementary_Data [file qyaf079_supplementary_data.docx]
